# The anti-HER3 antibody patritumab abrogates cetuximab resistance mediated by heregulin in colorectal cancer cells

**DOI:** 10.18632/oncotarget.2663

**Published:** 2014-12-05

**Authors:** Hisato Kawakami, Isamu Okamoto, Kimio Yonesaka, Kunio Okamoto, Kiyoko Shibata, Yume Shinkai, Haruka Sakamoto, Michiko Kitano, Takao Tamura, Kazuto Nishio, Kazuhiko Nakagawa

**Affiliations:** ^1^ Department of Medical Oncology, Kinki University Faculty of Medicine, Osaka-sayama, Osaka 589-8511, Japan; ^2^ Center for Clinical and Translational Research, Kyushu University Hospital, Higashiku, Fukuoka 812–8582, Japan; ^3^ Department of Genome Biology, Kinki University Faculty of Medicine, Osaka-Sayama, Osaka 589–8511, Japan

**Keywords:** colorectal cancer, heregulin, resistance, cetuximab, patritumab

## Abstract

We previously showed that tumor-derived heregulin, a ligand for HER3, is associated with both de novo and acquired resistance to cetuximab. We have now examined whether patritumab, a novel neutralizing monoclonal antibody to HER3, is able to overcome such resistance. Human colorectal cancer (DiFi) cells that are highly sensitive to cetuximab were engineered to stably express heregulin by retroviral infection, and the effects of cetuximab and patritumab on the resulting DiFi-HRG cells were examined. DiFi-HRG cells released substantial amounts of heregulin and showed resistance to cetuximab. Cetuximab alone inhibited EGFR and ERK phosphorylation in DiFi-HRG cells, but it had no effect on the phosphorylation of HER2, HER3, or AKT, suggesting that sustained AKT activation by HER2 and HER3 underlies cetuximab resistance in these cells. In contrast, patritumab in combination with cetuximab markedly inhibited the phosphorylation of EGFR, HER2, HER3, ERK, and AKT. The combination therapy also inhibited the growth of DiFi-HRG tumor xenografts in nude mice to a greater extent than did treatment with either drug alone. Activation of HER2-HER3 signaling associated with the operation of a heregulin autocrine loop confers resistance to cetuximab, and patritumab is able to restore cetuximab sensitivity through inhibition of heregulin-induced HER3 activation.

## INTRODUCTION

Cetuximab, a chimeric human-mouse monoclonal antibody to the epidermal growth factor receptor (EGFR), has shown clinical efficacy in individuals with metastatic colorectal cancer (mCRC). However, a subset of mCRC patients fails to show an initial response (de novo resistance) to this agent, whereas others develop resistance after an initial response (acquired resistance). Well-established causes of de novo resistance to cetuximab include activating mutations in codon 12 or 13 of *KRAS* and in *BRAF* [1–4]. Various mechanisms responsible for acquired resistance to cetuximab in colorectal cancer have also been identified [5–7]. We previously established cetuximab-resistant cancer cells by exposing parental cells to increasing concentrations of cetuximab [8]. Analysis of these cells revealed that cell-derived heregulin confers cetuximab resistance through bypass signaling via HER2 (also known as ERBB2) and HER3 (also known as ERBB3). Heregulin is a ligand for HER3 and stabilizes the HER2-HER3 heterodimer [9]. We also found that high initial levels of serum heregulin protein and tumor heregulin mRNA were significantly associated with a poor clinical outcome in mCRC patients treated with cetuximab [8]. Furthermore, in patients who initially achieved a partial response to cetuximab-based therapy, the serum concentration of heregulin after the development of clinical cetuximab resistance was significantly higher than that before treatment [8]. These preclinical and clinical data indicate that increased levels of heregulin are associated with both de novo and acquired resistance to cetuximab.

Patritumab (U3-1287) is a first-in-class, fully human monoclonal antibody directed to the extracellular domain (ECD) of HER3 that is currently in clinical development, as are other HER3-targeted antibodies such as MM-121 and LJM716 (MM-121 prevents ligand binding, whereas LJM716 specifically binds to an epitope formed by ECD domains II and IV in the closed conformation of HER3 [10]). Patritumab has been shown both to inhibit ligand-induced HER3 phosphorylation and to suppress the growth of pancreatic, non–small cell lung cancer, and colorectal cancer xenograft tumors [11, 12]. To identify strategies or agents capable of overcoming resistance to cetuximab induced by heregulin, we have now established sublines of the cetuximab-sensitive human colorectal cancer cell line DiFi that stably express heregulin derived from transfected cDNA. With the use of these cells, we investigated the effects of patritumab on cetuximab resistance mediated by cell-derived heregulin both *in vitro* and *in vivo*.

## RESULTS

### DiFi cells stably overexpressing heregulin show resistance to cetuximab

**Figure 1:**
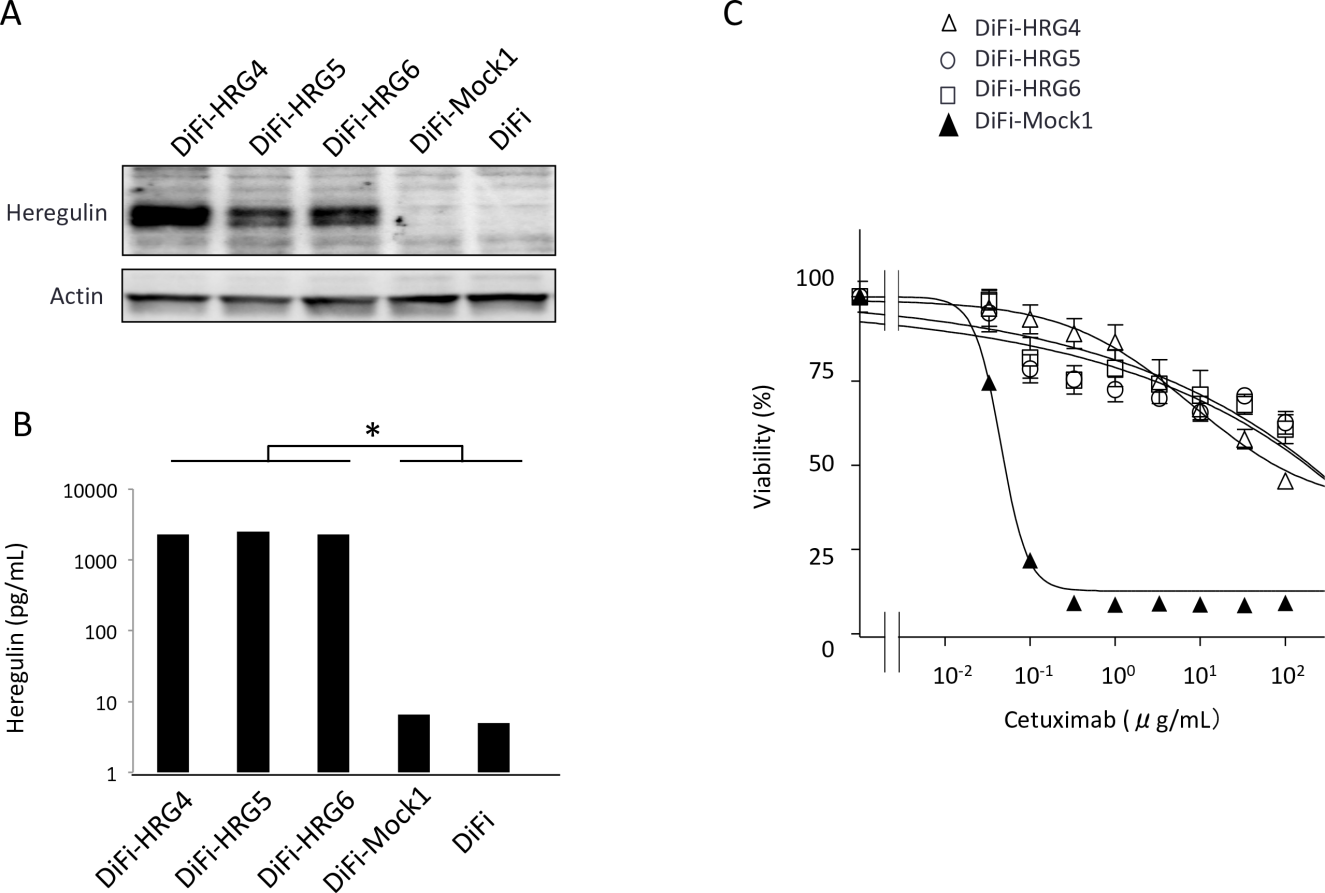
Characterization of DiFi isogenic cell lines **(A)** DiFi isogenic cell lines (DiFi, DiFi-Mock1, DiFi-HRG4, DiFi-HRG5, and DiFi-HRG6) were cultured overnight in medium containing 10% serum and then incubated for 24 h in serum-free medium, after which the cells were lysed and subjected to immunoblot analysis with antibodies to heregulin and to β-actin (loading control). **(B)** Culture supernatants from cells cultured as described in Materials and Methods were assayed for heregulin with an ELISA. Data are means ± SE from three independent experiments. *P < 0.05 (Student's *t* test) for comparison of each DiFi-HRG line with DiFi-Mock1 or DiFi cells. **(C)** Cells were treated with cetuximab at the indicated concentrations for 5 days, after which cell viability was assessed. Data are means ± SE from three independent experiments.

The human colorectal cancer cell line DiFi, which harbors wild-type alleles of *KRAS, BRAF*, and *PI3K*, is highly sensitive to cetuximab [13]. To investigate whether cell-derived heregulin might induce cetuximab resistance in DiFi cells, we established DiFi sublines that stably overexpress this protein (DiFi-HRG4, DiFi-HRG5, and DiFi-HRG6) or that stably harbor the corresponding empty vector (DiFi-Mock1) as a result of retroviral infection. Heregulin is a soluble growth factor that is synthesized as a transmembrane precursor molecule of 105 kDa. Cell surface proteases catalyze cleavage of the extracellular domain of this precursor, which is then released and functions as a ligand for HER3. Immunoblot analysis revealed the presence of the transmembrane form of heregulin in DiFi-HRG cells (with its abundance being greatest in DiFi-HRG4 cells), whereas no such band was detected in DiFi-Mock1 cells or the parental DiFi cells (Fig. 1A). Analysis of conditioned medium from these cell lines with an enzyme-linked immunosorbent assay (ELISA) also revealed the presence of substantial amounts of heregulin in the medium from all DiFi-HRG cell lines but not in that from DiFi-Mock1 or the parental cells (Fig. 1B). To assess the effect of cetuximab on cell growth, we exposed DiFi-HRG and DiFi-Mock1 cells to various concentrations of the drug for 5 days and then measured cell viability. All DiFi-HRG cell lines showed a reduced sensitivity to cetuximab compared with DiFi-Mock1 cells, with median inhibitory concentration (IC_50_) values of > 100 μg/mL for the former cell lines and ~0.1 μg/mL for the latter (Fig. 1C). The DiFi-HRG cell lines also showed resistance to panitumumab, another antibody to EGFR (data not shown). These data thus suggested that DiFi-HRG cells are resistant to EGFR-targeted antibodies.

### Heregulin maintains HER3 and AKT phosphorylation and survivin expression in the presence of cetuximab in DiFi-HRG cell lines

**Figure 2:**
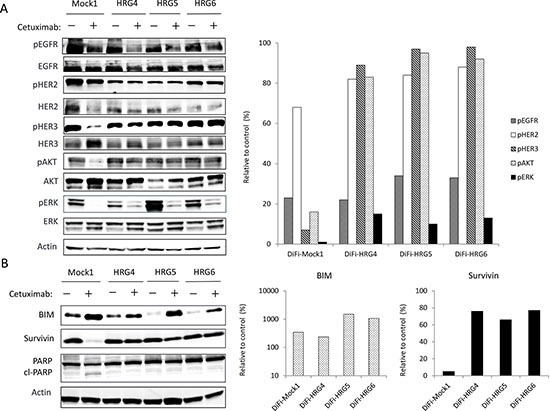
Effects of cetuximab on intracellular signaling and the expression of apoptosis-related proteins in DiFi isogenic cell lines DiFi-Mock1, DiFi-HRG4, DiFi-HRG5, or DiFi-HRG6 cells were cultured overnight in medium containing 10% serum and then incubated for 6 h **(A)** or 24 h **(B)** in serum-free medium with or without cetuximab (10 μg/mL), after which cell lysates were prepared and subjected to immunoblot analysis with antibodies to phosphorylated (p) or total forms of the indicated proteins (left panels). A band corresponding to the cleaved (cl) form of PARP is indicated. The intensity of the bands corresponding to phosphorylated forms of EGFR, HER2, HER3, AKT, and ERK (A) or to BIM and survivin (B) was normalized by that of the corresponding total proteins or β-actin, respectively, and then expressed relative to the corresponding value for control cells not exposed to cetuximab (right panels).

To investigate possible differences in signal transduction among the DiFi isogenic lines, we examined the effects of cetuximab (10 μg/mL) on EGFR, HER2, HER3, AKT, and extracellular signal–regulated kinase (ERK) phosphorylation (Fig. 2A). Immunoblot analysis revealed that cetuximab markedly inhibited the phosphorylation of all of these proteins in DiFi-Mock1 cells. In contrast, whereas cetuximab substantially reduced the level of EGFR and ERK phosphorylation in DiFi-HRG cells, it had little effect on the phosphorylation of HER2, HER3, or AKT. We next examined the effects of cetuximab on expression of the apoptosis-related proteins BIM (a proapoptotic BH3-only protein) and survivin (a member of the inhibitor of apoptosis, or IAP, family). We previously showed that inhibition of the MEK-ERK signaling pathway induces BIM expression, and that inhibition of the PI3K-AKT pathway suppresses survivin expression, with both of these effects being independently required for tyrosine kinase inhibitor (TKI)–induced apoptosis in lung cancer cells positive for *EGFR* mutation [14], breast cancer cells positive for *HER2* amplification [15], and gastric cancer cells positive for *MET* amplification [16]. Consistent with these observations, we found that cetuximab induced both up-regulation of BIM and down-regulation of survivin in DiFi-Mock1 cells, resulting in generation of the cleaved form of poly(ADP-ribose) polymerase (PARP), a characteristic of apoptosis (Fig. 2B). In contrast, in DiFi-HRG cell lines, whereas cetuximab induced BIM expression, it had little effect on the abundance of survivin or PARP cleavage (Fig. 2B), suggesting that sustained AKT signaling and survivin expression confer resistance to cetuximab in these cell lines.

### The HER3 neutralizing antibody patritumab abrogates cetuximab resistance induced by heregulin

**Figure 3:**
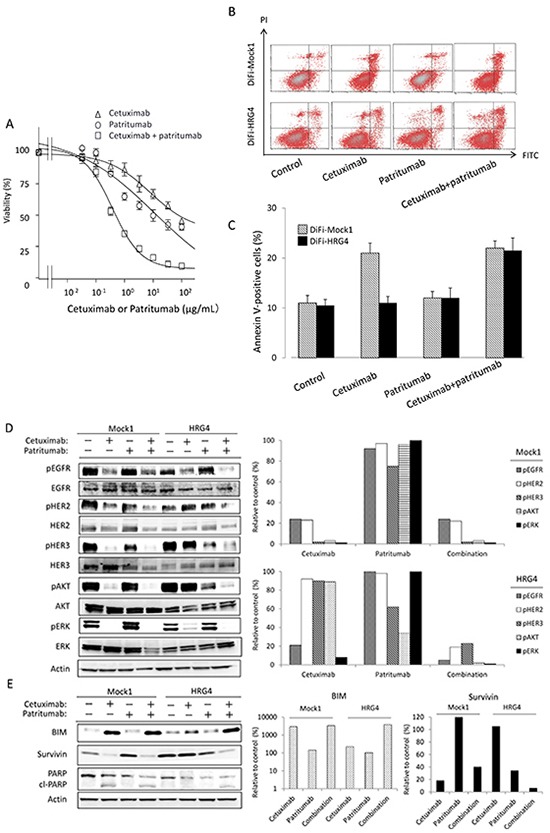
Effect of patritumab on heregulin-mediated cetuximab resistance in DiFi-HRG cells *in vitro* **(A)** DiFi-HRG4 cells were incubated for 5 days with cetuximab alone, patritumab alone, or the combination of both drugs at the indicated concentrations, after which cell viability was assessed. Data are means ± SE from three independent experiments. **(B, C)** DiFi-Mock1 or DiFi-HRG4 cells were cultured overnight in medium containing 10% serum and then incubated for 48 h in the absence or presence of cetuximab alone (10 μg/mL), patritumab alone (10 μg/mL), or the combination of both drugs in serum-free medium, after which the number of apoptotic cells was determined by staining with propidium iodide (PI) and fluorescein isothiocyanate (FITC)–labeled annexin V followed by flow cytometry. Representative flow cytometric profiles are shown in (B), and quantitative data (means ± SE of three independent experiments) are shown in (C). **(D, E)** DiFi-Mock1 or DiFi-HRG4 cells were cultured overnight in medium containing 10% serum and then incubated for 6 h (D) or 48 h (E) in the absence or presence of cetuximab alone (10 μg/mL), patritumab alone (10 μg/mL), or the combination of both drugs in serum-free medium, after which cell lysates were prepared and subjected to immunoblot analysis with antibodies to the indicated proteins (left panels). The intensity of the bands corresponding to phosphorylated forms of EGFR, HER2, HER3, AKT, and ERK (D) or to BIM and survivin (E) was normalized by that of the corresponding total proteins or β-actin, respectively, and then expressed relative to the corresponding value for control cells not exposed to drug (right panels).

To investigate further the role of HER3 and heregulin in the resistance of DiFi-HRG cell lines to cetuximab, we exposed DiFi-HRG4 cells to cetuximab, the fully human HER3-targeted monoclonal antibody patritumab, or the combination of both agents. We found that neither antibody alone substantially affected cell proliferation, whereas the combination of both agents induced marked inhibition of cell growth (Fig. 3A). We next examined the effects of these antibodies on apoptosis in DiFi-Mock1 and DiFi-HRG4 cells. An annexin V binding assay revealed that cetuximab alone induced a substantial level of apoptosis in DiFi-Mock1 cells but not in DiFi-HRG4 cells (Fig. 3B, C), suggesting that the operation of a heregulin autocrine loop in these latter cells inhibits cetuximab-induced apoptosis. However, exposure of DiFi-HRG4 cells to the combination of patritumab (10 μg/mL) and cetuximab (10 μg/mL) resulted in a marked increase in the proportion of apoptotic cells (Fig. 3B, C), suggesting that patritumab sensitizes DiFi-HRG cells to cetuximab such that the extent of apoptosis induced by both antibodies in these cells is similar to that induced by cetuximab alone in DiFi-Mock1 cells.

We also examined the effects of patritumab alone or in combination with cetuximab on intracellular signaling. Immunoblot analysis showed that patritumab alone had little effect on such signaling in DiFi-Mock1 cells. In contrast, patritumab alone markedly inhibited the phosphorylation of HER3 and AKT, without affecting that of ERK, in DiFi-HRG4 cells (Fig. 3D). The combination of patritumab and cetuximab markedly attenuated the phosphorylation of EGFR, HER2, HER3, AKT, and ERK in DiFi-HRG4 cells (Fig. 3D). It also induced the cleavage of PARP in these cells to an extent similar to that observed in DiFi-Mock1 cells treated with cetuximab alone, and this effect was accompanied by both up-regulation of BIM and down-regulation of survivin expression (Fig. 3E). These results thus indicated that cetuximab resistance induced by heregulin is abrogated by patritumab through attenuation of AKT-suvivin signaling in DiFi-HRG4 cells.

### Cell-derived heregulin induces cetuximab resistance and patritumab restores cetuximab sensitivity in tumor xenografts *in vivo*

**Figure 4:**
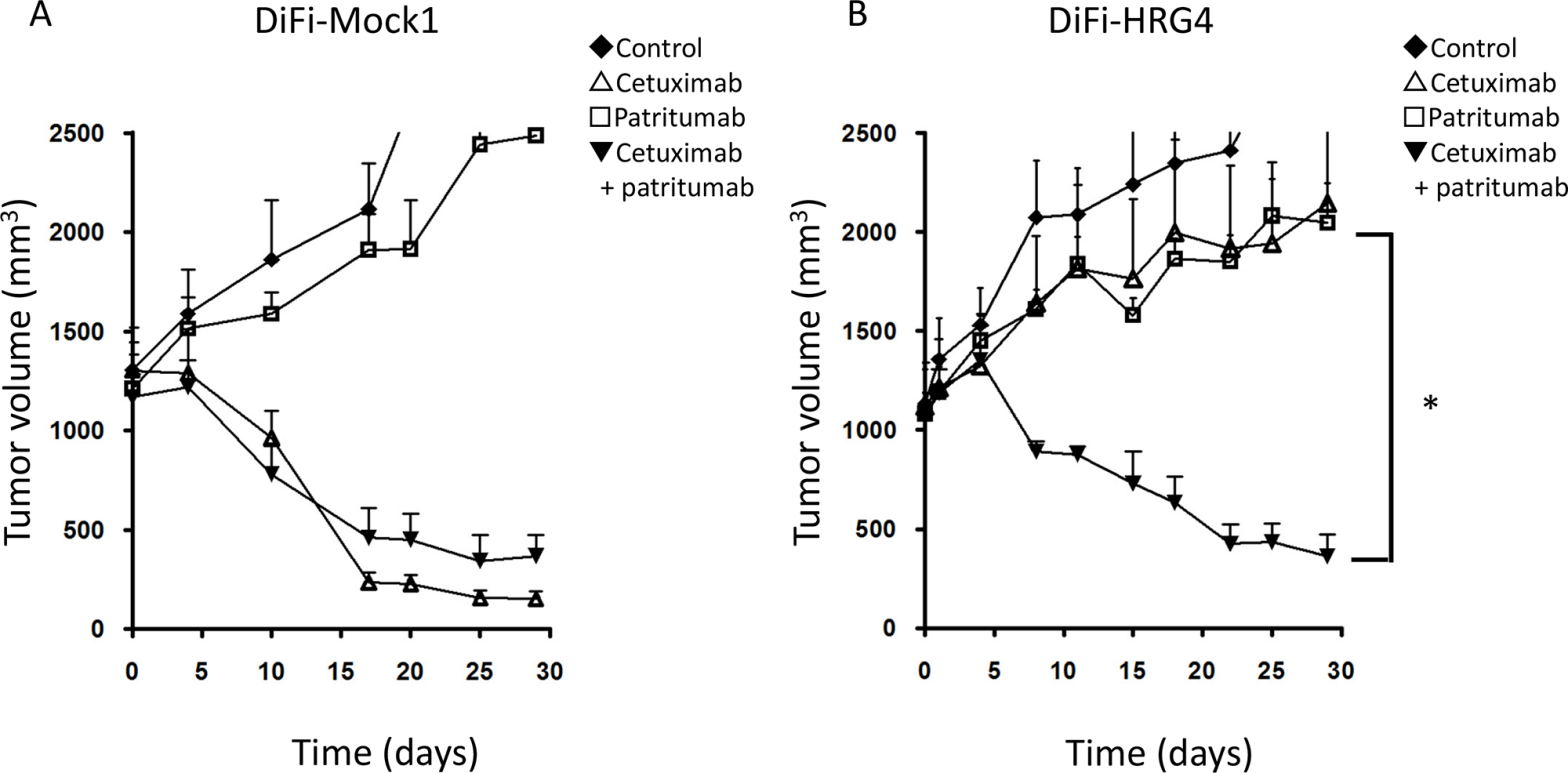
Effects of cetuximab, patritumab, and the combination of both drugs on the growth of DiFi-HRG tumor xenografts *in vivo* Nude mice with tumor xenografts established by subcutaneous injection of DiFi-Mock1 **(A)** or DiFi-HRG4 **(B)** cells were treated for 4 weeks with vehicle (control), cetuximab (1.0 mg/body), patritumab (1.0 mg/body), or both drugs, as described in Materials and Methods. Tumor volume was determined at the indicated times after the onset of treatment. Data are means ± SE from six mice per group. *P < 0.05 for comparison of the combination of both drugs with cetuximab alone or patritumab alone (Student's *t* test).

To examine whether cell-derived heregulin induces cetuximab resistance as well as the efficacy of combined treatment with patritumab and cetuximab *in vivo*, we injected nude mice with DiFi-Mock1 or DiFi-HRG4 cells to allow the formation of tumor xenografts. Whereas cetuximab alone markedly inhibited the growth of DiFi-Mock1 xenografts (Fig. 4A), DiFi-HRG4 xenografts were resistant to this drug (Fig. 4B). Patritumab alone had little effect on the growth of tumors formed by either cell line. However, the combination of cetuximab and patritumab induced substantial regression of DiFi-HRG4 xenografts (Fig. 4B). These results thus suggested that heregulin produced by colorectal cancer tumors harboring wild-type *KRAS* induces cetuximab resistance, and that combination therapy with cetuximab and patritumab overcomes such resistance *in vivo*.

## DISCUSSION

Resistance to cetuximab is a major problem in the treatment of colorectal cancer. Although various mechanisms of cetuximab resistance have been identified [1–7, 17–20], the optimal treatment strategies for mCRC patients who show resistance to this drug remain unclear. We previously showed that tumor-derived heregulin mediates cetuximab resistance in preclinical models [8]. High levels of heregulin were also associated with a poor clinical outcome in mCRC patients treated with cetuximab-based regimens [8]. Moreover, increased heregulin levels were observed in such patients after the development of clinical resistance to cetuximab-based therapy [8]. Effective treatment options to overcome cetuximab resistance mediated by heregulin are thus urgently required.

We have here established heregulin-overexpressing sublines of DiFi cells (DiFi-HRG cells) and shown that these cells are resistant to cetuximab both *in vitro* and *in vivo*. Whereas the amount of the transmembrane form of heregulin was substantially higher in DiFi-HRG4 cells than in DiFi-HRG5 or DiFi-HRG6 cells (Fig. 1A), these three cell lines appeared to release similar amounts of the soluble form of heregulin into the culture medium (Fig. 1B). This difference in the relative abundance of the transmembrane and soluble forms of heregulin among DiFi-HRG cell lines might reflect a difference in the activity of cell surface proteases among the cell lines. Alternatively, the production of the transmembrane form of heregulin in DiFi-HRG4 cells might exceed the capacity of such proteases. To investigate the mechanism responsible for cetuximab resistance in DiFi-HRG cells, we examined differences in signal transduction between these cells and DiFi-Mock1 cells. In DiFi-Mock1 cells, cetuximab inhibited the phosphorylation of EGFR, HER2, HER3, AKT, and ERK as well as up-regulated BIM expression and down-regulated survivin expression, resulting in the induction of apoptosis. By contrast, in DiFi-HRG cell lines, whereas cetuximab inhibited EGFR and ERK phosphorylation, leading to BIM induction, it did not affect HER2, HER3, or AKT phosphorylation or survivin expression. Given that down-regulation both of AKT signaling and of the expression of its downstream target survivin is required for apoptosis induced by inhibition of receptor tyrosine kinases [14–16], our data suggest that sustained AKT-survivin signaling in the presence of cetuximab is responsible for the resistance of DiFi-HRG cell lines to this drug. To investigate further the relation between AKT signaling and the operation of a heregulin autocrine loop, we examined the effects of patritumab, a neutralizing monoclonal antibody to HER3, in DiFi-HRG4 cells. We found that exposure of these cells to patritumab in combination with cetuximab resulted in inhibition of EGFR, HER2, HER3, AKT, and ERK phosphorylation as well as in both up-regulation of BIM expression and down-regulation of survivin expression, leading to the induction of apoptosis. These results indicate that AKT signaling is triggered by heregulin binding to HER3.

**Figure 5:**
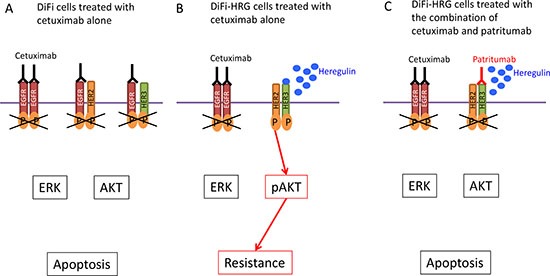
Model for intracellular signaling underlying the induction of apoptosis by cetuximab and patritumab in colorectal cancer cells **(A)** DiFi colorectal cancer cells treated with cetuximab alone. **(B)** DiFi cells that stably overexpress heregulin (DiFi-HRG cells) are resistant to cetuximab as a result of HER2-HER3 heterodimerization and AKT activation induced by heregulin. **(C)** Patritumab abrogates cetuximab resistance mediated by the heregulin autocrine loop in DiFi-HRG cells.

Given that HER3 (a kinase-dead receptor) manifests impaired kinase activity [21], it requires dimerization with other HER family members to activate signaling after ligand binding [22, 23]. HER2 has been implicated as a dimerization partner of HER3, and heregulin stabilizes the HER2-HER3 heterodimer [9]. In this context, we examined the effects of lapatinib, a TKI for EGFR and HER2, in DiFi-HRG4 cells. Inhibition of EGFR and HER2 by lapatinib resulted in down-regulation of HER3, AKT, and ERK phosphorylation as well as in the induction of BIM and suppression of survivin expression in these cells, thereby triggering apoptosis (Supplementary Fig. S1A, S1B). These findings suggest that, in DiFi-HRG cells, HER3 is trans-phosphorylated by HER2 as a result of heregulin-induced HER2-HER3 heterodimerization, which in turn leads to the activation of AKT signaling [24] (Fig. 5A, 5B, 5C). The heterodimerization partner of HER3 is thus likely switched from EGFR to HER2 as a result of the overexpression of heregulin in DiFi-HRG cells. We also found that patritumab alone inhibited the phosphorylation of HER3 and AKT as well as down-regulated survivin expression in DiFi-HRG4 cells but not in DiFi-Mock1 cells (Fig. 3D, 3E). These results suggest that patritumab may prevent ligand-dependent HER3 phosphorylation by blocking HER2-HER3 heterodimerization, whereas it has little effect on ligand-independent HER3 phosphorylation.

In our DiFi-HRG xenograft model, we showed that combination therapy with patritumab and cetuximab inhibited tumor growth to the same extent as did cetuximab alone in the DiFi-Mock1 xenograft model. Antagonism of the heregulin-HER3 interaction by patritumab thus represents an effective strategy to abrogate cetuximab resistance induced by heregulin derived from tumor cells. Given that elevated circulating levels of heregulin are associated with both de novo and acquired cetuximab resistance in mCRC patients [8], our model systems based on stable overexpression of heregulin are clinically relevant and should prove useful for establishing strategies to overcome cetuximab resistance mediated by the heregulin autocrine loop. Indeed, a recent phase I/II study with refractory colorectal cancer patients revealed potential antitumor activity of the combination of cetuximab and pertuzumab, a HER2-targeted antibody that blocks ligand-dependent HER2-HER3 heterodimerization. However, this drug combination was not tolerable as a result of overlapping toxicities [25]. Given that the toxicity profile of patritumab differs from that of pertuzumab [26], the combination of cetuximab and patritumab warrants evaluation in the clinical setting. We also found that the IC_50_ value for the antiproliferative effect of the combination of cetuximab and patritumab in DiFi-HRG4 cells was ~10 times as high as that for cetuximab alone in DiFi-Mock1 cells (Fig. 1C, Fig. 3A). The discrepancy between these *in vitro* data and our *in vivo* findings may suggest that antibody-dependent cellular cytotoxicity [27] involving NK cell activation plays a role in tumor growth inhibition by the combination of both agents. Moreover, combination therapy with these two IgG1 antibodies may result in an enhanced antitumor activity mediated by cytotoxic T lymphocytes [28] in the clinical setting. Given the recent evidence implicating the importance of interactions between therapeutic antibodies and the immune system in the efficacy of antibody treatment [20], further investigation of such mechanisms is warranted.

In conclusion, we have shown that consecutive activation of HER2-HER3 and AKT by heregulin in an autocrine-dependent manner confers resistance to cetuximab, and that patritumab restores cetuximab sensitivity in tumors with heregulin-induced cetuximab resistance. Further studies of combination therapy with patritumab and cetuximab are thus warranted in mCRC patients with heregulin-induced resistance to EGFR-targeted antibodies.

## MATERIALS AND METHODS

### Cells and reagents

The DiFi human colorectal cancer cell line was kindly provided by P. A. Janne (Dana Farber Cancer Institute). DiFi cells were maintained under a humidified atmosphere of 5% CO_2_ in air at 37°C in Dulbecco's modified Eagle's medium (DMEM) containing high glucose and supplemented with Ham's F-12 and 10% fetal bovine serum (FBS). Cetuximab was obtained from Merck Serono, and patritumab was kindly provided by Daiichi-Sankyo (Tokyo, Japan). Recombinant human heregulin (NRG1-β1/HRG1-β1 extracellular domain) was obtained from R&D Systems.

### Establishment of cells stably overexpressing heregulin

A full-length cDNA encoding human heregulin (NRG1, GenBank accession no. NM_013956) was obtained from Origene (Rockville, MD). The amplification product was verified by sequencing after its cloning into the pCR-Blunt II-TOPO vector (Invitrogen). The heregulin cDNA was then excised from pCR-Blunt II-TOPO and transferred to the pQCXIH retroviral vector (Clontech), and retroviruses encoding heregulin were produced and used to infect DiFi cells as described [29]. Cells stably expressing heregulin were then isolated by selection with hygromycin (Invivogen) at 500 μg/mL.

### Cell growth inhibition assay

Cells were transferred to 96-well flat-bottomed plates and cultured for 24 h before exposure to various concentrations of cetuximab or patritumab in medium containing 1% FBS for 120 h. Cell Counting Kit-8 solution (Dojindo, Kumamoto, Japan) was then added to each well, and the cells were incubated for 3 h at 37°C before measurement of absorbance at 490 nm with a Multiskan Spectrum instrument (Thermo Labsystems). Absorbance values were expressed as a percentage of that for nontreated cells, and the IC_50_ of cetuximab for inhibition of cell growth was determined.

### Heregulin ELISA

The concentration of heregulin in cell culture supernatants was measured with the use of a sandwich ELISA (NRG1-β1 DuoSet, R&D Systems) as previously described [30]. Cells were seeded in six-well plates at a density of 0.5 × 10^6^ cells per well in DMEM supplemented with 10% FBS. After the cells had achieved confluence, the medium was replaced with 5 ml of DMEM supplemented with 0.1% FBS, the cells were incubated for 48 h, and the culture supernatants were collected for assay of heregulin.

### Immunoblot analysis

Cells were washed twice with ice-cold phosphate-buffered saline (PBS) and then lysed with 1 × Cell Lysis Buffer (Cell Signaling Technology) consisting of 20 mmol/L Tris-HCl (pH 7.5), 150 mmol/L NaCl, 1 mmol/L EDTA (disodium salt), 1 mmol/L EGTA, 1% Triton X-100, 2.5 mmol/L sodium pyrophosphate, 1 mmol/L β-glycerophosphate, 1 mmol/L Na_3_VO_4_, leupeptin (1 μg/mL), and 1 mmol/L phenylmethylsulfonyl fluoride. The protein concentration of the lysates was determined with a bicinchoninic acid assay kit (Thermo Fisher Scientific), and equal amounts of protein were subjected to SDS-polyacrylamide gel electrophoresis on a 7.5% gel (Bio-Rad). The separated proteins were transferred to a nitrocellulose membrane, which was then incubated with Blocking One solution (Nacalai Tesque) for 20 min at room temperature before incubation overnight at 4°C with primary antibodies. Antibodies to heregulin (NRG1-β1), to phosphorylated EGFR (phospho-Tyr^1068^), to phosphorylated HER2 (phospho-Tyr^1248^), to phosphorylated HER3 (phospho-Tyr^1289^), to phosphorylated or total forms of AKT, to phosphorylated ERK, to PARP, and to BIM were obtained from Cell Signaling Technology; those to total HER3, to total ERK, and to survivin were from Santa Cruz Biotechnology; those to total EGFR were from Zymed/Invitrogen; those to total HER2 were from Millipore; and those to β-actin were from Sigma. The membrane was then washed with PBS containing 0.05% Tween 20 before incubation for 1 h at room temperature with horseradish peroxidase–conjugated secondary antibodies (GE Healthcare). Immune complexes were finally detected with ECL detection reagents (GE Healthcare).

### Annexin V binding assay

The binding of annexin V to cells was measured with the use of an Annexin-V-FLUOS Staining Kit (Roche). Cells were harvested by exposure to trypsin-EDTA, washed with PBS, and centrifuged at 200 × *g* for 5 min. The cell pellets were resuspended in 100 μL of Annexin-V-FLUOS labeling solution, incubated for 10 to 15 min at 15° to 25°C, and then analyzed for fluorescence with a flow cytometer (FACSCalibur) and Cell Quest software (Becton Dickinson).

### Tumor growth inhibition assay *in vivo*

All animal experiments were performed in accordance with the Recommendations for Handling of Laboratory Animals for Biomedical Research compiled by the Committee on Safety and Ethical Handling Regulations for Laboratory Animal Experiments, Kinki University. The ethical procedures followed conformed to the UKCCCR guidelines for the welfare and use of animals in cancer research [31]. The study was also reviewed and approved by the Animal Ethics Committee of Kinki University (approval no. KAME-22-018). Cells were injected subcutaneously into the axilla of 5- to 6-week-old female athymic nude mice (BALB/c nu/nu; CLEA Japan), and treatment was initiated when tumors in each group of six mice achieved an average volume of 1000 to 1200 mm^3^. Treatment groups consisted of vehicle control, cetuximab (1.0 mg/body), patritumab (1.0 mg/body), and the combination of both agents. Cetuximab and patritumab were administered by intraperitoneal injection twice a week for 4 weeks, with control animals receiving a 0.5% (w/v) aqueous solution of hydroxyl propylmethyl cellulose as vehicle. Tumor volume was determined from caliper measurements of tumor length (*L*) and width (*W*) according to the formula *LW *^2^/2. Both tumor size and body weight were measured twice weekly. For ethical reasons, animals were removed from the study if the tumor volume exceeded 2500 mm^3^.

### Statistical analysis

Quantitative data are presented as means ± SE unless indicated otherwise. The significance of differences was evaluated with the unpaired two-tailed Student's *t* test. A *P* value of < 0.05 was considered statistically significant.

## SUPPLEMENTARY FIGURE


