# Supplementary material for: The anti-HER3 antibody patritumab abrogates cetuximab resistance mediated by heregulin in colorectal cancer cells
Source: Oncotarget. 2014 Dec 5;5(23):11847–56. doi: 10.18632/oncotarget.2663 (PMC4323007; doi:10.18632/oncotarget.2663)
Supplement: Supplementary file 1 [file oncotarget-05-11847-s001.pdf]

SUPPLEMENTARY FIGURE

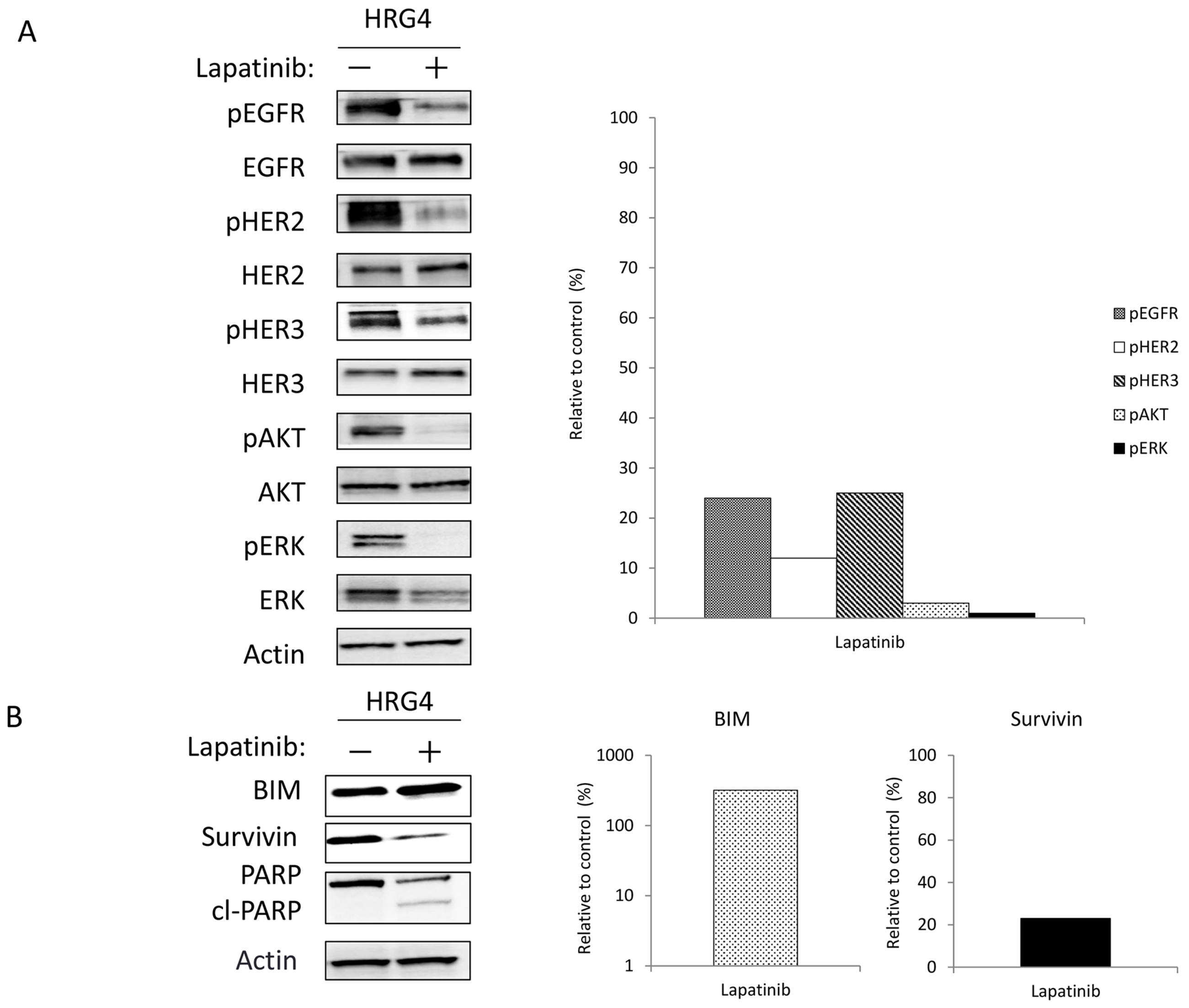

Supplementary Figure S1: Effects of lapatinib on intracellular signaling in DiFi-HRG cells. DiFi-HRG4 cells were cultured overnight in medium containing 10% serum and then incubated for 6 h **(A)** or 48 h **(B)** in serum-free medium with or without lapatinib (1 μmol/L), after which cell lysates were prepared and subjected to immunoblot analysis with antibodies to the indicated proteins (left panels). The intensity of the bands corresponding to phosphorylated forms of EGFR, HER2, HER3, AKT, and ERK **(A)** or to BIM and survivin **(B)** was normalized by that of the corresponding total proteins or β-actin, respectively, and then expressed relative to the corresponding value for control cells not exposed to drug (right panels).
